# A Rare Complication of Fine-Needle Aspiration of Neck Structures

**DOI:** 10.1155/2021/8944119

**Published:** 2021-12-15

**Authors:** Yazeed M. Qadadha, Nainika Nanda, Chad Ennis, Timothy McCulloch

**Affiliations:** ^1^University of Wisconsin, School of Medicine and Public Health, 750 Highland Ave, Madison, WI 53726, USA; ^2^Division of Otolaryngology-Head and Neck Surgery, Department of Surgery, University of Wisconsin School of Medicine and Public Health, 600 Highland Avenue, Madison, WI 53792, USA

## Abstract

Fine-needle aspiration (FNA) is a generally accepted tool for safe diagnostic evaluation in the workup of lesions and masses. Aside from the commonly discussed risks of infection and minor bleeding related to skin puncture, other more serious complications have been reported sparingly. We present two cases of pneumothorax from FNA of neck structures, which have been theorized but not previously reported to our knowledge. Discussion of cases of this complication rather than solely a theoretical understanding of it will aid in diagnosis and management of this complication.

## 1. Introduction

Fine-needle aspiration (FNA) is a diagnostic procedure used to investigate lesions and masses in various parts of the body. It has gained significant utility in the head and neck region, including for evaluation of thyroid lesions, parotid lesions, and suspicious lymphadenopathy. Incorporating this tool in workup has been proven to be cost-effective, efficient, and generally with minimal risk [1]. The application of using a needle as a diagnostic and therapeutic approach for different conditions dates to the 900s [2]. FNA has applications in several superficial tissue types, including the breast, lymph nodes, thyroid, salivary glands, and soft tissues. Nonpalpable deeper lesions can also be biopsied using FNA with image guidance [1].

The benefits of FNA have been described using the acronym SAFE [3]. The procedure is generally considered *Simple* for the patient and provider, requiring no special preparation or dietary changes and only a few items to be handled by the specimen collector. FNAs collected by those who are experienced are usually *Accurate* and sufficient for diagnosis. The efficient collection process and rapid preliminary impression at resourced institutions have contributed to a *Fast* diagnosis and earlier treatment. By avoiding operating room and sedation or general anesthesia costs, FNA is considered significantly more *Economical* than more invasive diagnostic procedures or surgeries [3].

FNA risks are considered minimal in most settings [4]. With puncturing the skin, there is a small risk of bleeding, infection, and discomfort. Pain at the puncture site is usually well tolerated and may vary based on the underlying pathology [5]. Local pressure is usually sufficient for bleeding. With appropriate skin preparation, site infection is extremely unlikely [4, 6]. Complications of deep FNA biopsies (those of nonpalpable lesions) have been shown to be more common and more serious, such as severe bleeding and hematoma [1].

In addition to the uncommon risks of superficial biopsy discussed above, FNA of neck structures includes the rare risk of hematoma of the neck [7], seeding of cancer along the needle track [8], and acute bacterial suppurative thyroiditis [9]. Other risks have been theoretically speculated but have not, to our knowledge, been reported in the literature, including cyst fluid leakage, anaphylactic reaction, pneumothorax, and thromboembolism [10].

In this case series, we present two patients who underwent FNA of neck structures and suffered pneumothorax (PTX) as a complication. Pneumothorax (PTX) is defined as a volume of air entrapped in the thoracic cavity, between the visceral pleura and parietal pleura [11].

Iatrogenic pneumothorax has been reported in cases of pleural biopsy, transbronchial lung biopsy, transthoracic pulmonary nodule biopsy, central venous catheter insertion, tracheostomy, intercostal nerve block, and positive pressure ventilation [11]. To our knowledge, these are the first clinical scenarios to be described of iatrogenic pneumothorax resulting from neck FNA. Awareness of this potential complication will alert clinicians and help them keep PTX on the differential when caring for a patient with acute distress following FNA of the neck structures.

## 2. Case Report

**Case 1 .**

A 42-year-old female was referred to the otolaryngology clinic for thyroid FNA in the context of an intermittent hoarse dysphonia for several years along with sore throat and hair loss. She had a past medical history of temporomandibular joint disorder and cervical radiculopathy. She was a former tobacco smoker and quit in 2011. Her initial exam was notable for body habitus of a tall, athletic, Caucasian female. Workup by her PCP demonstrated a lesion below the left inferior thyroid lobe, prompting referral. Otherwise, thyroid function tests were within the reference range.

**Figure 1:**
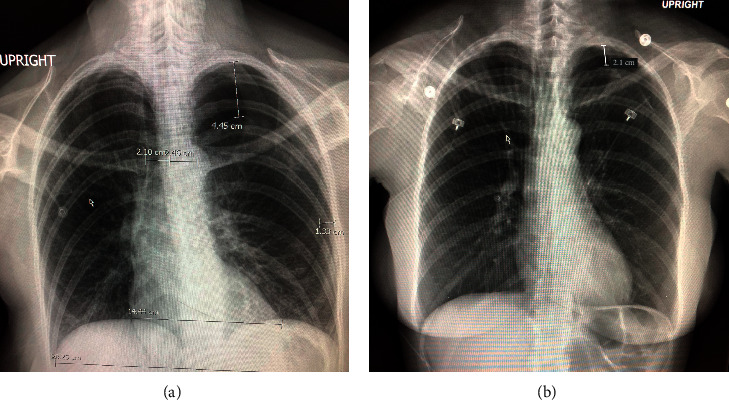
(a) Initial CXR in the emergency department showing a left pneumothorax measuring 4.45 cm separation of the pleura at the apex and 1.33 cm laterally. (b) Repeat CXR on postprocedure day 1 with 2.1 cm separation of pleura at the apex.

The target lesion was localized with ultrasound. Then, after administering local anesthetic superficial to the target lesion, an ultrasound-guided 22-gauge needle was used to obtain the specimen. The first pass caused pain at the biopsy site which was followed by delivery of additional local anesthetic over the same region. She also experienced a vasovagal response, which she reported had similarly occurred during previous procedures. Of note, preliminary cytology from FNA demonstrated colloid and follicular cells without evidence of malignancy. Shortly following completion of the third pass, she developed lightheadedness, diaphoresis, nausea, and progressive dyspnea and pain along the left chest wall. She was then transported to the emergency department (ED) for a chest X-ray (CXR) and further workup. ED evaluation was notable for the patient in tears due to left back pain and difficulty with deep inhalation. CXR was obtained and showed a left-sided apical pneumothorax (Figure 1(a)), and thoracic surgery was consulted. Repeat CXR in 1 hour demonstrated a decrease in PTX volume with pleural separation decreasing from 4.5 cm to 2.9 cm at the lung apex. The patient was then evaluated by thoracic surgery, and her exam was notable for stable vitals on room air and unlabored and even respirations with the lungs clear to auscultation throughout. The patient was then admitted for monitoring with telemetry and supplemental O_2_ without chest tube placement given her hemodynamic stability and improvement on repeat CXR in ED. The next morning (postprocedure day 1), the patient reported improvement in pain and was back on room air although reported residual pain with deep inspiration. Vitals remained within normal limits. Repeat CXR showed continued decrease in the volume of left apical PTX with separation down to 2.1 cm (Figure 1(b)). The patient was discharged home that day with recommendation of outpatient follow-up for 1 week. She presented to an outside hospital 10 days after discharge for follow-up and reported improving occasional discomfort and cough. Repeat CXR showed near-complete resolution of PTX, measuring about 4 mm of pleural separation. Repeat CXR in 1 more week demonstrated complete resolution of PTX.

**Case 2 .**

**Figure 2:**
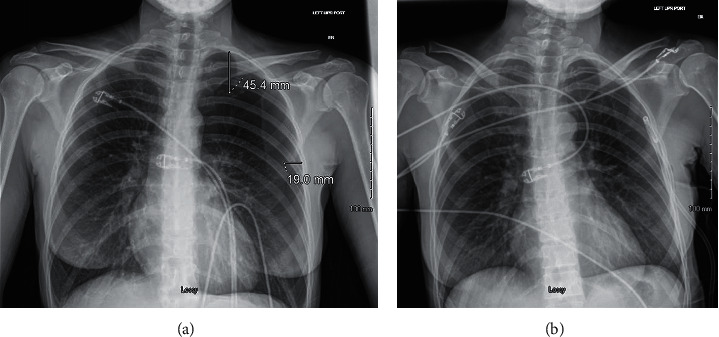
(a) Initial AP CXR showing medium left PTX measuring 4.54 cm separation of the pleura at the apex and 1.90 cm laterally. (b) Repeat AP CXR after chest tube placement with reexpansion of the left lung.

A 29-year-old female presented to the otolaryngology clinic after 2 months of enlarging left neck lymph nodes. She had no significant past medical history. Her social history was notable for 11 years of smoking, having quit in 2017. Recent CT with contrast identified multiple abnormally enlarged lymph nodes in the left lower neck/supraclavicular fossa. In the clinic, palpable mobile left level IV lymph nodes were confirmed by ultrasound. Local anesthesia was injected above the most medial lymph node, which was the one targeted for biopsy. She underwent an ultrasound-guided FNA using a 22-gauge needle where three samples were collected and lymphocytes were identified. A fourth sample was then taken for flow cytometry. All portions of the procedure were tolerated well. She was prescribed 2 weeks of Augmentin and sent home. On the way home, she developed substernal chest pain that worsened with deep inspiration, along with shortness of breath and a dry cough. She immediately returned to the ED, where, on exam, she appeared nontoxic with stable vital signs and decreased breath sounds in the left superior lung field. Her neck was nontender, and the trachea was midline. Initial CXR was notable for a medium left pneumothorax (Figure 2(a)). Given the size of the pneumothorax and her worsening symptoms, a 14-French chest tube was placed by ED providers with suction set to –20 cm·H_2_O. Repeat CXR was obtained after tube placement, which demonstrated decreased PTX volume (Figure 2(b)). She was admitted to the internal medicine service for iatrogenic PTX with pulmonary medicine consultation for chest tube management.

The patient remained hospitalized for 4 days with a fluctuating course. Early during her stay, she had persistence of a small apical pneumothorax, which was believed to be due to the tube not reaching the apex of her pleural space along with the presence of a positional, low-flow air leak. She failed a clamping trail on postprocedure day (PPD) 2, and a wall suction of −20 cm was restarted. On PPD3, suction was paused, and the chest tube was kept on water seal. After radiographic improvement, another clamping trial was attempted on PPD3 and failed. On PPD 4, the air leak resolved. She passed a clamping trial, and the chest tub was removed. She remained asymptomatic and had normal work of breathing after removal of the chest tube; therefore, she was discharged home. The patient was recommended to follow up with a primary care provider (PCP) for 5–7 days. Outside records show a follow-up CXR 11 days after discharge without an office visit. CXR was normal, with no evidence of recurrent pneumothorax. She returned to otolaryngology clinic 2 weeks after discharge to follow up on indeterminant FNA results and did not indicate any residual symptoms from the PTX. Of note, she ended up undergoing an open excisional biopsy, which was diagnostic of nodular sclerosis Hodgkin's lymphoma.

## 3. Discussion

In this series, we discuss iatrogenic pneumothorax in two patients following FNA biopsy of neck structures. To our knowledge, this is the first clinical scenario description of PTX related to head and neck FNA. In the first case, thyroid FNA was complicated by PTX with symptom onset immediately after the biopsy and managed conservatively. The second case describes a patient whose level IV lymph node FNA was complicated by PTX with symptom onset within hours of procedure completion and required a chest tube as part of management.

PTX can be classified as traumatic, atraumatic, or iatrogenic, depending on the mechanism by which air gains access to the pleural space [11]. Traumatic PTX can result from blunt or penetrating trauma. Atraumatic PTX is further divided into primary (rupture of bullae or blebs without a known reason) or secondary (related to underlying lung disease). PTX can also be categorized as simple, tension, or open. In simple PTX, there is no mediastinal shift, whereas in tension PTX, mediastinal shift results from air pressure accumulation due to air trapping in the pleural space in one hemithorax and compressing the contralateral side. Open PTX is characterized by an open chest wall wound allowing air movement into the chest cavity [11]. Prognosis is favorable for iatrogenic and traumatic PTX, in the absence of other severe injuries [12], while tension PTX or PTX from barotrauma has higher mortality [13].

In terms of iatrogenic PTX, rates have been between 21 and 60% in thoracic FNA [14]. This high rate has contributed to numerous studies in the field and to devising specific recommendations to decrease iatrogenic pneumothorax resulting from thoracic FNA. However, these recommendations have not been defined in the head and neck literature as readily due to the lack of reported cases. Modifiable risk factors may be mitigated by using a smaller-gauge needle, limiting the number of passes, and utilizing fluoroscopy to avoid blebs [14]. Nonmodifiable risk factors include obstructive and restrictive pulmonary diseases. Attempts to predict and quantify pneumothorax risk in patients with pulmonary diseases using spirometry have been made, but studies have shown mixed results regarding utility of spirometry for this objective [14–16]. The literature listing pneumothorax as a theoretical complication of neck FNA does offer suggestions to minimize this risk based on the understanding of pneumothorax in thoracic FNA [10]. These suggestions include using a 25-gauge needle or smaller when possible and performing ultrasound-guided rather than direct FNA, especially when targeting supraclavicular or deep-seated thyroid nodules [10]. In terms of diagnostic yield of FNA, some studies reported no difference when comparing head and neck specimens obtained with a 21-gauge needle and a 25-gauge needle [17], or between 23-gauge needle and a 27-gauge needle [18]. Another study looking at thyroid FNA showed that biopsies using 25-gauge needles have a higher yield than those using 22-gauge needles [19]. Ultrasound guidance was utilized in both of our cases. In each of our cases, a 22-gauge needle was used which may have slightly increased the risk. Neither one of our patients had any known underlying pulmonary pathology. The history of smoking can also increase risk for a primary spontaneous pneumothorax but has not been shown to affect the risk of iatrogenic pneumothorax [11]. A pneumothorax shortly after an FNA is more likely to be related to the procedure than coincidence.

Even though our patient in “Case 1” showed clear signs of distress, she did not exhibit typical symptoms of a clinically significant pneumothorax until shortly after, such as dyspnea, tachypnea, chest pain, or pleurisy [20]. Pneumothorax management ranges from conservative management and observation to time-sensitive decompression depending on the clinical scenario. Needle decompression is the appropriate treatment for unstable patients. Chest imaging should not delay needle decompression in such cases. Following a needle decompression or in stable pneumothorax, a chest tube is inserted to prevent the pressure from building back up in the pleural space. Asymptomatic patients with small primary spontaneous pneumothorax with a depth less than 2 cm can be discharged with a follow-up of 2–4 weeks as outpatients [11]. An asymptomatic patient with small secondary spontaneous pneumothorax with a depth less than 1 cm should be admitted and started on high flow oxygen and observed for 24 hours [11]. If the depth is 1-2 cm, a needle aspiration is performed, and imaging is repeated to assess the residual pneumothorax size [11]. If residual depth is less than 1 cm, supplemental oxygen and observation are usually sufficient [11]. If the residual depth is greater than 2 cm or in the case of continued dyspnea, tube thoracostomy should be performed [11]. In iatrogenic cases, pneumothoracies greater than 20% of the hemithorax volume or those producing symptoms should be evacuated with a chest tube and patients should be admitted for continued observation [20]. Those less than 20% which are also asymptomatic and hemodynamically stable can be managed with supplemental oxygen and observation with repeat imaging at 12 or 24 hours or with changes in symptoms. Stable patients with resolving pneumothorax on repeat imaging may be discharged. Stable patients with unchanged imaging findings may also be discharged with plan to follow-up in 48 hours. If reimaging shows progression or if the patient becomes symptomatic, evacuation of the pneumothorax is required [20, 21]. Therefore, the decision to adopt the conservative approach of oxygen supplementation and observation with our patient in Case 1 was made by considering her hemodynamic stability, normal oxygenation on room air, and quick improvement in the volume of PTX on CXR. In our second case, the decision to insert a chest tube was appropriate given the worsening symptoms of dyspnea, chest pain, and pleurisy [20]. Her hemodynamic stability was the reason why a chest tube was more appropriate than needle decompression [11].

The cases presented demonstrate the importance of considering pneumothorax in patients presenting in distress following FNA biopsy of the neck. This should also encourage all clinicians to actively minimize the risk by using smaller-size needles and continuing to utilize ultrasound guidance. Gaining familiarity with potential complications will facilitate patient counseling and timely diagnosis, decreasing morbidity and mortality.

## 4. Conclusion

Fine-needle aspiration is a relatively safe and effective diagnostic tool. Clinicians performing FNA of neck structures should be aware of all potential complications, including pneumothorax, to incorporate that into patient counseling, monitoring, and management. Incidence of pneumothorax in neck FNA is unknown due to the paucity of literature, but care must be taken to minimize the risk of this potentially morbid complication.

## Data Availability

As this is a case report on patients' clinical progress, data collection was unnecessary. Further imaging details are available from the corresponding author upon request.
